# Vascular endothelial growth factor (VEGFA) gene variation in polycystic ovary syndrome in a Tunisian women population

**DOI:** 10.1186/s12864-016-3092-5

**Published:** 2016-10-17

**Authors:** Assila Ben Salem, Fatma Megdich, Olfa Kacem, Malek Souayeh, Faten Hachani Ben Ali, Sondes Hizem, Faouzi Janhai, Mounir Ajina, Muhammad Abu-Elmagd, Mourad Assidi, Mohammed H. Al Qahtani, Touhami Mahjoub

**Affiliations:** 1 Laboratory of Human Genome and multifactorial diseases, LR12ES07, Faculty of Pharmacy of Monastir, University of Monastir, Monastir, Tunisia; 2 Department of Gyneco Obstetric, University Hospital F. Hached, Sousse, Tunisia; 3 University Hospital F. Hached, Unit of Reproductive Medicine, Sousse, Tunisia; 4 Center of Excellence in Genomic Medicine Research (CEGMR), King Abdulaziz University, Jeddah, Saudi Arabia

**Keywords:** PCOS, VEGFA, SNP, Haplotype, Prolactin, Genetic association, Polymorphism

## Abstract

**Background:**

Polycystic ovary syndrome (PCOS) is characterized by the growth of a number of small cysts on the ovaries which leads to sex hormonal imbalance. Women who are affected by this syndrome suffer from irregular menstrual cycles, decline in their fertility, excessive hair growth, obesity, acne and most importantly cardiac function problems. The vascular endothelial growth factor (VEGF) plays a pivotal role in tissue vascularization in general and in the pathogenesis of many diseases. The PCOS was found to be associated with high expression levels of VEGF. In women who undergo assisted reproductive procedures (ART), VEGF was found to be a key mediator of other factors to control ovary angiogenesis. Here, we set out to examine the association of VEGFA gene polymorphism with PCOS and its components in a population of Tunisia women to enhance our understanding of the genetic background leading angiogenesis and vascularization abnormalities in PCOS.

**Methods:**

The association of VEGFA gene with PCOS and its components was examined in a cohort of 268 women from Tunisia involving 118 PCOS patients and 150 controls. VEGFA gene variations were assessed through the analysis of the following SNPs rs699947 (A/C), rs833061 (C/T), rs1570360 (G/A), rs833068 (G/A), rs3025020 (C/T), and rs3025039 (C/T). The linkage disequilibrium between SNPs was assessed using HAPLOVIEW software while combination of SNPs into haplotypes in the population and the reconstruction of the cladogram were carried-out by PHASE and ARLEQUIN programs, respectively. Genetic association and genotype-phenotype correlations were calculated by logistic regression and non-parametric tests (Kruskall-Wallis and Mann–Whitney tests), respectively, using StatView program.

**Results:**

We observed 10 haplotypes in our studied cohort whereH1 (ACGG), H2 (ACAG), H7 (CTGG) and H8 (CTGA) were the most frequent. We observed the association of the genotype CT of the SNP rs30225039 with PCOS phenotype (*P* = 0.03; OR 95 % CI = 2.05 [1.07–3.90]) and a trend for correlation of the pair of haplotypes H2/H2 with prolactin levels in plasma (*P* = 0.077; 193.5 ± 94.3 vs 45.7 ± 7.2). These data are consistent with literature and highlight one more time the role of vascularization in the pathogeny of PCOS.

**Conclusions:**

LD pattern in VEGF locus showed a similar LD pattern between the Tunisian population and the CEU. More haplotypes in the Tunisian population than in CEU was observed (22 haplotypes vs 16 haplotypes) suggesting higher recombination rate in Tunisians. The study showed that there was any advantage of using haplotypes compared with SNPs taken alone.

## Background

The polycystic ovary syndrome (PCOS) is a disease affecting 6 – 12 % of women at reproductive age and characterized by abnormalities in relation to reproduction, hyperandrogenism as well as metabolic abnormalities like insulin resistance, obesity, impaired fasting glucose (IGF) and metabolic syndrome (MetS) [1, 2].

Knowing the role of blood flow and vascular pattern of an organ in the organ’s morphology and function, previous studies focused on the comparison of blood flow and vascularization between women with PCOS and healthy women showing larger and more vascularized ovaries in PCOS women compared to the control [3]. Moreover, abnormalities in ovarian angiogenesis were involved in the ovarian hyperstimulation syndrome (OHSS) and other disorders of anovulation, subfertility and pathogenic conditions as endometriosis [4]. In this context, the vascular endothelial growth factor (VEGF) system, composed of ligands and receptors plays a pivotal role in tissue vascularization and endothelial cell growth [5]. It was also reported to induce cell proliferation, promote cell migration, inhibit apoptosis and induce permeabilization of blood vessels. It is a major component of the family of angiogenic factors, which includes placental growth factor, angiopoetin, basic fibroblast growth factor and the VEGF family (A, B, C, D and E). VEGFA in particular is a protein encoded by a gene (Gene ID: 7422) located in chromosome 6 and extended on more than 16 kb (GRCh37/hg19; chr6: 43737946–43754224). The gene involves 8 exons and encodes for many VEGFA isoforms as VEGF-165 and VEGF-145.

Previous studies reported the association of VEGFA gene with PCOS through SNPs +9812, +13553, −2578 (rs699947), −460 (rs833061) and +405 [6, 7] and their haplotypes in populations of different origins [6–8]. Moreover, SNPs +405 and −460 were reported as having a trend towards higher insulin resistance [9]. It was suggested the role of some of these SNPs on the regulation of VEGFA gene expression and protein production and secretion [10].

In this context, we focused on the study of the association of VEGFA gene with PCOS and its components in a population of women from Tunisia well characterized at the phenotypic level. The aim was to better characterize the genetic background responsible for angiogenesis and vascularization abnormalities in PCOS. This will be achieved through testing the association of VEGFA gene in a population where it was previously highlighted the need for haplotyping approach in order to understand better the genetic association in populations with mosaic anthropogenetic components [11, 12].

### Subjects and methods

1. Subjects
  Following an informed consent, a total of 268 unrelated Tunisian women, consisting of 118 PCOS patients and 150 controls. PCOS patients group (mean age: 29.8 ± 0.4 years) were recruited from the outpatient Obstetrics and Gynecology Department, CHU Farhat Hached (Sousse) in Central Tunisia (Table 2). The diagnosis of PCOS was based on the 2003 Rotterdam Criteria [13]. PCOS was then attested when two of the following three conditions are observed: anovulation, hyperandrogenism, and the presence of polycystic ovaries on ultrasound examination. Exclusion criteria were androgen-producing tumors, 21-hydroxylase-deficiency, non-classical adrenal hyperplasia, hyperprolactinemia, active thyroid disease, and Cushing’s syndrome.
  Control group consisted of 150 healthy women (mean age: 33.5 ± 0.5 years), with regular menstrual cycles and no evidence of hirsutism, acne, alopecia, or endocrinopathies. None of the controls was on hormonal therapy (including oral contraceptives) for the previous three months or longer, and none of PCOS women or controls was on medication known to affect carbohydrate metabolism or endocrine parameters, at least for the last three months before inclusion in the study. Demographic data and history of hypertension, diabetes, and hypercholesterolemia were recorded for all subjects. Obesity was defined as body-mass index (BMI) ≥30 kg/m^2^. The protocol of the study was approved by the local ethics committee, and written informed consent was obtained from all subjects.
2. SNPs genotyping
  Total genomic DNA was isolated from peripheral blood lymphocytes by the salting-out method. Six SNPs were selected because of their association with PCOS, angiogenesis and other metabolic components in literature: rs699947 (A/C), rs833061 (C/T), rs1570360 (G/A), rs833068 (G/A), rs3025020 (C/T), andrs3025039 (C/T). The 6 SNPs were genotyped by the allelic discrimination TaqMan SNP Genotyping Assays (*Applied Biosystems; Foster City, CA*). The PCR primers and TaqManprobes were directly available from Applied Biosystem (predesigned assays) and analyses were done according to the manufacturer’s protocol on a StepOne plus apparatus (Applied Biosystems) (Table 1).

**Table 1:** SNPs probe sequence

SNP	Assay ID	Probe sequence
rs699947	C_8311602_10	GCCAGCTGTAGGCCAGACCCTGGCA[A/C]GATCTGGGTGGATAATCAGACTGAC
rs833061	C_1647381_10	GAGTGTGTGCGTGTGGGGTTGAGGG[C/T]GTTGGAGCGGGGAGAAGGCCAGGGG
rs1570360	C_1647379_10	AGCCCGGGCCCGAGCCGCGTGTGGA[A/G]GGGCTGAGGCTCGCCTGTCCCCGCC
rs833068	C_11400864_10	GACATGTCCCATTTGTGGGAACTGT[A/G]ACCCTTCCTGTGTGAGCTGGAGGCA
rs3025020	C_1647366_10	GCCTCTGGAGGGGAGCCCCCTATTC[C/T]GGCCCAACCCATGGCACCCACAGAG
rs3025039	C_16198794_10	GCATTCCCGGGCGGGTGACCCAGCA[C/T]GGTCCCTCTTGGAATTGGATTCGCC

### Statistical analysis

Data were expressed as mean ± SE (continuous variables) or as percent of total (categorical variables), and intergroup significance was assessed by *χ*2 test. Allele frequencies were calculated by gene-counting method; each SNP was tested for Hardy–Weinberg equilibrium using Haploview 4.2 (http://www.broadinstitute.org/haploview). Linkage disequilibrium (LD) was assessed using Haploview 4.2. Genetic association and genotype-phenotype correlation were assessed by logistic regression and non-parametric tests (Mann–Whitney and Kruskall-Walli tests) using StatView program.

Calculation of the power of the study was assessed using SAMPSIZE software (http://sampsize.sourceforge.net).

## Results and discussions

### Phenotype data

Women with PCOS have higher BMI than controls (*P* < 0.0001; 28.4 ± 0.7 vs 23.1 ± 0.2 kg/m^2^), higher random glycemia (*P* <0.0001, 7.9 ± 0.2 vs 4.5 ± 0.1 mmol/L), fasting insulin (*P* = 0.01, 15.7 ± 1.2vs 7.7 ± 0.4 mU/L), levels of total testosterone (*P* < 0.0001, 2.9 ± 0.2 vs 1.0 ± 0.1 noml/L) and of plasma triglycerides (TG) (*P* = 0.0001, 1.7 ± 0.1 vs 1.0 ± 0.1). Prolactin levels in plasma were lower in women with PCOS compared with controls (*P* = 0.0001, 73.1 ± 11.7 vs 148.8 ± 9.4) (Table 2).

**Table 2:** Phenotypic features of our women cohort used in the study

	PCOS women (*n* = 118)	Controls (*n* = 150)	*P* (*X* ^*2*^)^a^
Age (years)	29.8 ± 0.4	33.5 ± 0.5	<0.0001
BMI (kg/m^2^)	28.4 ± 0.7	23.1 ± 0.2	<0.0001
Random glycemia (mmol/L)	7.9 ± 0.2	4.5 ± 0.1	<0.0001
Fasting insulin (mU/L)	15.7 ± 1.2	7.7 ± 0.4	0.01
Testosterone (nmol/L)	2.9 ± 0.2	1.0 ± 0.1	<0.0001
Prolactin (mU/L)	73.1 ± 11.7	148.8 ± 9.4	0.0001
Triglycerides (mmol/L)	1.7 ± 0.1	1.0 ± 0.1	0.0001
LDL cholesterol	3.1 ± 0.1	1.9 ± 0.2	<0.0001
MetS ATPIII (%)	34.7	0.0	<0.0001

^a^The significance of the difference between cases and controls is assessed by *X* ^2^ test

### Genotype data

All SNPs were in Hardy-Weinberg equilibrium. Allele frequencies of SNPs in PCOS and controls were respectively0.56 and 0.56 for rs699947, 0.51 and 0.54 for rs833061, 0.34 and 0.31 for rs1570360, 0.37 and 0.36 for rs833068, 0.25 and 0.22 for rs3025020 and 0.87 and 0.91 for rs3025039 (Table 3).

**Table 3:** Allele frequencies of SNPs in PCOS and controls

SNP	Position	Minor Allele	Allele frequencies		*P* ^a^
			PCOS	Controls	
rs699947	43736389	A	0.56	0.56	0.873
rs833061	43737486	C	0.51	0.54	0.656
rs1570360	43737830	A	0.34	0.31	0.520
rs833068	43742527	A	0.37	0.36	0.820
rs3025020	43749110	T	0.25	0.22	0.403
rs3025039	43752536	C	0.87	0.91	0.126

^a^Pearson’s chi square test

**Fig. 1:**
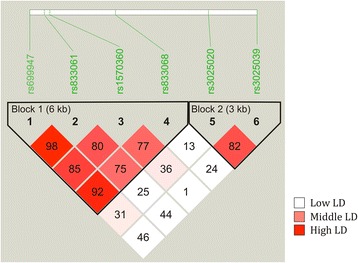
Linkage disequilibrium (LD) pattern in the locus of VEGFA gene delimited by SNPs rs699947 and rs3025039. Numbers in the squares indicate D’ index (level of LD) between the corresponding SNPs

The study of LD pattern (assessed by D’ index) in the region delimited by these SNPs showed relative high LD between SNPs rs699947, rs833061, rs1570360 and rs833068 (Fig. 1) allowing then reconstruction of haplotypes with these 4 polymorphisms. We observed 10 haplotypes among which 4 haplotypes were the most frequent: H1 (ACGG)(13.4 %), H2 (ACAG) (29.3 %), H7 (CTGG) (19.6 %) and H8 (CTGA) (32.3 %)(Table 4).

**Table 4:** List of haplotypes resulting from the combination of SNPs rs699947, rs833061, rs1570360 and rs833068 in the Tunisian population

Haplotype	rs699947 (A/C)	rs833061 (C/T)	rs1570360 (G/A)	rs833068 (G/A)	Prevalence (%)	*P* (*χ* ^2^)
H1	A	C	G	G	13.4	0.749
H2	A	C	A	G	29.3	0.595
H3	A	C	A	A	0.9	0.021
H4	A	T	A	G	0.4	0.081
H5	C	C	G	G	0.2	0.059
H6	C	C	G	A	2.1	0.781
H7	C	T	G	G	19.6	0.725
H8	C	T	G	A	32.3	0.389
H9	C	T	A	G	0.4	0.068
H10	C	T	A	A	1.5	0.088

H1 is the ancestral haplotype (haplotype of chimpanzee: Pan troglodytes)

P (*χ* ^2^) indicates the significance of variability of each polymorphism between the two groups

*NS* not statistically significant

**Fig. 2:**
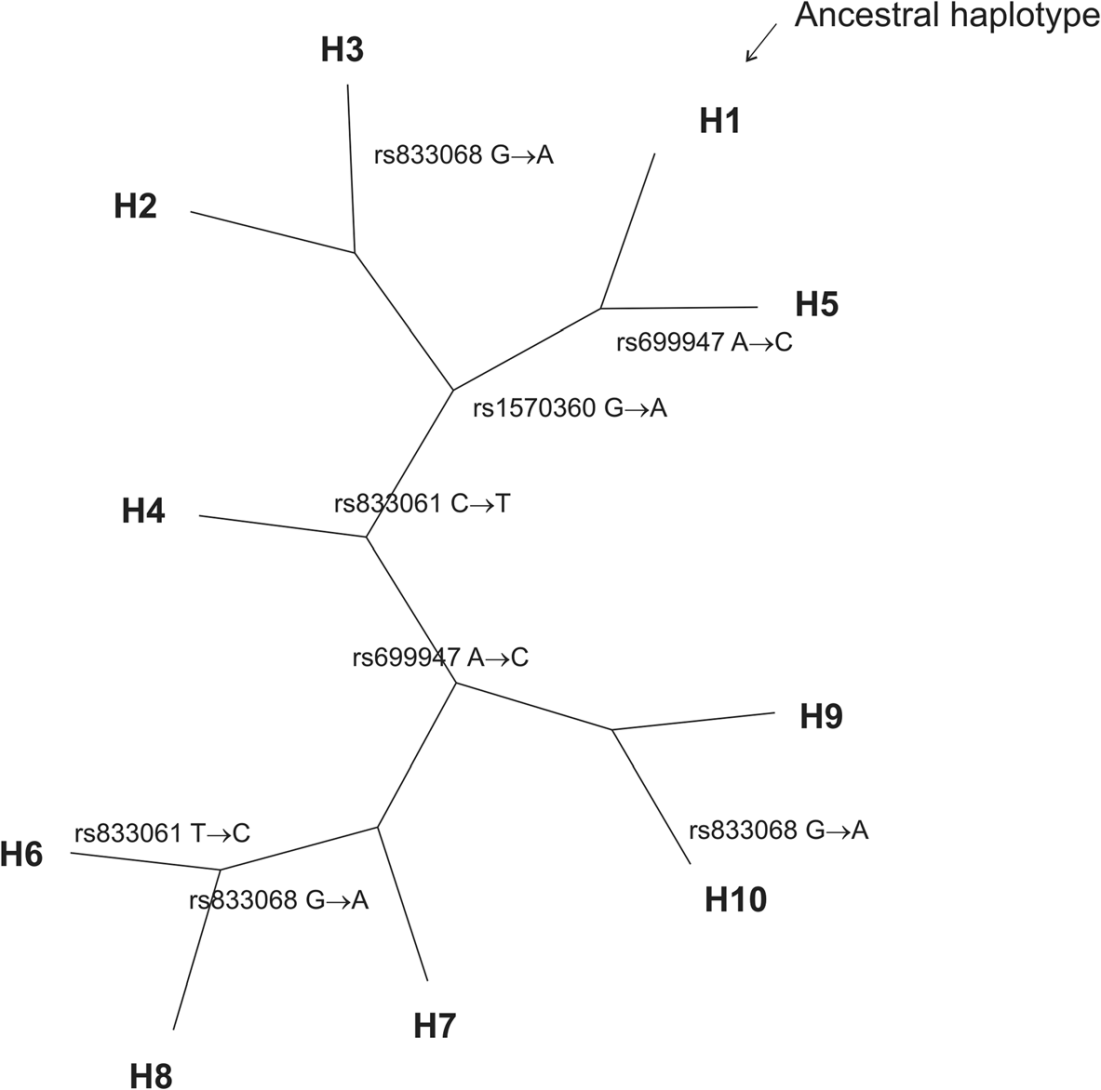
Cladistic representation of VEGFA haplotypes in the studied population. SNPs mutations responsible for the transition between haplotypes are indicated in the cladogram. H1 is the ancestral haplotype

Reconstruction of the cladogram in the population allowed observing that H2 arose from H1 after the mutation of the SNP r1570360 while H7 appeared after the mutation of rs1570360, rs833061 and rs699947. Finally, H8 arose after the mutation of rs1570360, rs833061, rs699947 and rs833068 (Fig. 2).

Logistic regression showed the association of the genotype CT of rs30225039 with PCOS phenotype (*P* = 0.03; OR 95 % CI = 2.05 [1.07 – 3.90]) (Table 5) while non-parametric tests allowed observing a trend for correlation of the pair of haplotypes H2/H2 to prolactin levels in plasma (*P* = 0.077; 193.5 ± 94.3 vs 45.7 ± 7.2). According to the calculation, this study had a power of 58.38 %. PCOS is a disease that affects women at reproductive age and characterized by endocrine disorders as menses disturbance and subfertility and by metabolic abnormalities as insulin resistance, and glucose intolerance [14, 15]. Angiogenesis process has also been reported as probably implied in disorders of anovulation, subfertility and other pathogenic conditions as endometriosis. Since VEGF system plays a pivotal role in tissue vascularization, VEGFA gene is considered as candidate in reproductive abnormalities as PCOS. Many studies have been carried-out and concluded in a part of them that VEGFA levels in serum or follicular fluid are higher in women with PCOS [16–19]. In another hand, previous studies in Korean and Turkish populations reported the association of VEGFA gene with PCOS through many SNPs and one haplotype [6–8]. In that context, our investigation focused on the study of VEGFA gene association with PCOS in a Tunisian population well characterized at the phenotypic level, through 6 SNPs and their combination into haplotypes in the population. We observed the association of the gene (SNP rs30225039) with PCOS phenotype and a trend of correlation (pair of haplotypes H2/H2) with prolactin levels in plasma. Prolactin is secreted mainly by the pituitary gland and is implied in many processes as lactation and also tissue vascularization [20]. Indeed, many studies showed the role of prolactin as a pro-angiogenic factor while its enzymatically cleaved 16 kDa N-terminal fragment has a well-defined anti-angiogenic effect [21]. Moreover, it was shown the effect of prolactin on the induction of VEGF expression [22–24]. To the best of our knowledge, our study is the first work that reports the correlation, even with a trend, of VEGFA gene variations with prolactin plasma levels. These findings suggest possible regulation and/or feedback control of prolactin secretion pathway through VEGF and the role of VEGFA gene variation in this process. Deeper genetic investigations taking into account more SNPs and larger locus are necessary to demystify these molecular pathways.

**Table 5:** Distribution of VEGF genotype in PCOS cases and control women

	1/1^a^		1/2^a^		2/2^a^		
	PCOS	Controls	PCOS	Controls	PCOS	Controls	*P*
rs699947	0.3^b^	0.3	0.53	0.51	0.17	0.19	0.65
rs833061	0.28	0.28	0.46	0.51	0.25	0.21	0.38
rs1570360	0.48	0.5	0.36	0.38	0.16	0.12	0.71
rs833068	0.36	0.43	0.53	0.42	0.11	0.15	0.09
rs3025020	0.58	0.6	0.34	0.35	0.08	0.05	0.38
rs3025039	0.75	0.85	0.23	0.13	0.02	0.03	0.03

^a^Genotypes were coded as per “1” = major allele, “2” = minor allele

^b^Frequency

**Fig. 3:**
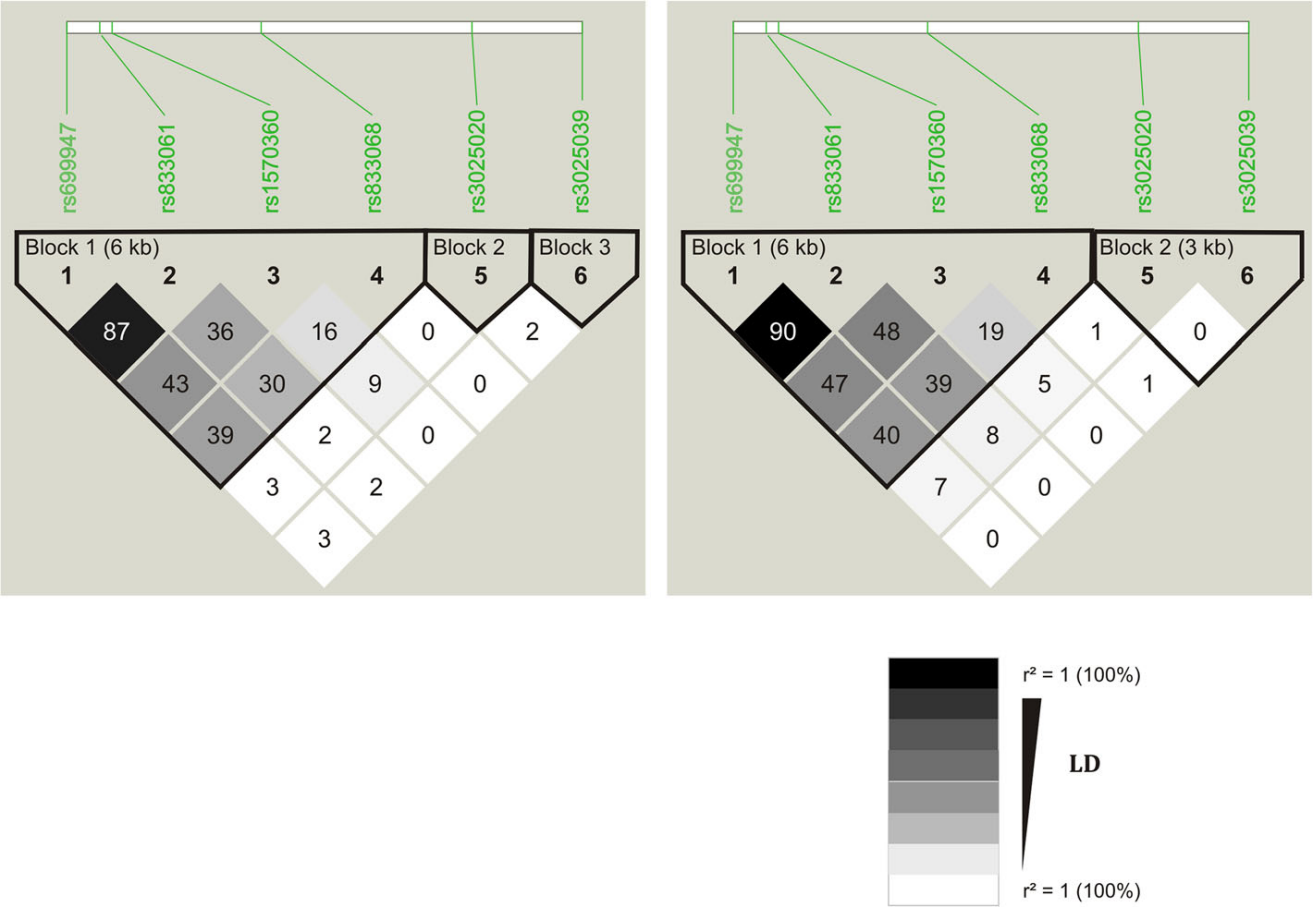
Comparison of linkage disequilibrium (LD) pattern (LD is assessed by r^2^ index) in the VEGF locus delimited between SNPs rs699947 and rs3025039, between the studied women population and the CEU reference population. Numbers in the squares indicates levels of LD as informed by r^2^ index

Differences of VEGF plasma levels between PCOS and healthy women were observed in some populations but not in others and this could be due to genetic differences between ethnic groups [16–19] Studying VEGFA gene association in complex disorders should take into account the gene variability in the population through the LD pattern assessment and SNPs combination into haplotypes. We reported in a previous study the role of haplotypes as powerful genetic markers for better characterization of gene association in the Tunisian population and observed Fat mass and obesity associated (FTO) gene association with PCOS through haplotypes but not through SNPs taken alone [12]. Indeed, we observed LD pattern in the FTO locus less in the Tunisian population compared with the CEU Caucasian one justifying so the ability of haplotypes for detecting genetic association in such conditions. In the present study, the investigation of the LD pattern in VEGF locus showed a similar LD pattern between the Tunisian population and the CEU one (Fig. 3). However, we found more haplotypes in the Tunisian population than in CEU (22 haplotypes vs 16 haplotypes) suggesting more recombination rate operated in Tunisians. Although we were not able to observe any advantage of using haplotypes compared with SNPs taken alone in this study, these results could suggest a more complex anthropogenetics context concerning VEGF locus or the need for a better characterization of this locus by using more density of SNPs at larger genomic region/scale and using larger cohorts of patients. Therefore, better management approaches can be identified [25]. Additionally, and despite the relatively reduced size of our cohort, it remains that the powering of the study was acceptable (58.38 %) attesting then for the reliability of our findings.

## Conclusions

Deeper investigations of VEGFA gene encompassing larger regions in the locus and using more SNPs need to be envisioned in order to better characterize the gene association in the Tunisian population and to shed light on the genetic/genomic players involved and develop better diagnostic, management and therapeutic approaches.
